# Molecular cloning, characterisation and molecular modelling of two novel T-synthases from mollusc origin

**DOI:** 10.1093/glycob/cwae013

**Published:** 2024-02-17

**Authors:** Marilica Zemkollari, Chris Oostenbrink, Reingard Grabherr, Erika Staudacher

**Affiliations:** Department of Chemistry, University of Natural Resources and Life Sciences, Muthgasse 18, 1190 Vienna, Austria; Department of Material Sciences and Process Engineering, University of Natural Resources and Life Sciences, Muthgasse 18, 1190 Vienna, Austria; Department of Biotechnology, University of Natural Resources and Life Sciences, Muthgasse 18, 1190 Vienna, Austria; Department of Chemistry, University of Natural Resources and Life Sciences, Muthgasse 18, 1190 Vienna, Austria

**Keywords:** β-1, 3-galactosyltransferase, glycobiology, molluscs, O-glycosylation, T-synthase

## Abstract

The glycoprotein-N-acetylgalactosamine β1,3-galactosyltransferase, known as T-synthase (EC 2.4.1.122), plays a crucial role in the synthesis of the T-antigen, which is the core 1 O-glycan structure. This enzyme transfers galactose from UDP-Gal to GalNAc-Ser/Thr. The T-antigen has significant functions in animal development, immune response, and recognition processes. Molluscs are a successful group of animals that inhabit various environments, such as freshwater, marine, and terrestrial habitats. They serve important roles in ecosystems as filter feeders and decomposers but can also be pests in agriculture and intermediate hosts for human and cattle parasites. The identification and characterization of novel carbohydrate active enzymes, such as T-synthase, can aid in the understanding of molluscan glycosylation abilities and their adaptation and survival abilities. Here, the T-synthase enzymes from the snail *Pomacea canaliculata* and the oyster *Crassostrea gigas* are identified, cloned, expressed, and characterized, with a focus on structural elucidation. The synthesized enzymes display core 1 β1,3-galactosyltransferase activity using pNP-α-GalNAc as substrate and exhibit similar biochemical parameters as previously characterised T-synthases from other species. While the enzyme from *C. gigas* shares the same structural parameters with the other enzymes characterised so far, the T-synthase from *P. canaliculata* lacks the consensus sequence CCSD, which was previously considered indispensable.

## Introduction

O-glycans are sugar chains that are attached to serine or threonine residues within a polypeptide sequence via an O-glycosidic bond. Among the various forms of O-glycosylation, mucin-type O-glycosylation has been extensively studied in mammals and is composed of eight core structures (Spiro  2002; Brockhausen  et al.  2022) However, in invertebrates, *Drosophila melanogaster* and snails, mainly core 1 and its variations have been identified (Stepan  et al.  2012; Zhang  and  Ten  Hagen  2019). The process of mucin-type O-glycosylation starts with the addition of GalNAc by ppGalNAc transferases (Bennett  et al.  2012). The glycan chain is then elongated by adding GalNAc, GlcNAc, or Gal residues. The glycoprotein-N-acetylgalactosamine β-1,3-galactosyltransferase transfers Gal from UDP-Gal to the GalNAc bound to the polypeptide chain, producing the core 1 O-glycan or T-antigen. In mammals, the folding and activity of this enzyme depends on a molecular chaperone called Cosmc (core 1 β1,3 galactosyltransferase specific molecular chaperone), but no such chaperone has been identified in invertebrates yet (Ju  et al.  2006; Yoshida  et al.  2008; Morio  et al.  2023; Zemkollari  et al.  2023). Changes in the activity of this enzyme result in a shorter core 1 O-glycan called the Tn-antigen. The presence of this structure has been linked to several human autoimmune diseases (Ju  and  Cummings  2005; Suzuki  et al.  2011), involvement in tumour progression (Huang  et al.  2014; Wan  and  Yu  2021; Xia  et al.  2022) and incorrect animal development (Xia  et al.  2004; Tran  and  Ten  Hagen  2013). Despite the very important biological roles of O-linked glycans, achieving a comprehensive understanding of their structures and enzymes involved in their biosynthesis remains a challenge, especially in molluscs. Molluscs represent one of the most diverse groups of animal kingdom with an estimated number of 100,000 species, around 80% of them are gastropods (Voronezhskaya  and  Croll  2015). With their unique characteristics, ecological significance and evolutionary adaptations, molluscs are a fascinating subject of scientific inquiry and a vital component of the world’s ecosystems. Many molluscs are filter feeders, helping to maintain water quality by filtering and removing particulate matter, and recycling nutrients. Some of them feed on plants which can result in substantial damage of crops and natural vegetation. Molluscs also serve as a vital food source for many other organisms, including fish, sea turtles, seabirds and also humans. Last but not least, many molluscs serve as intermediate hosts for many human and animal parasites.

*Pomacea canaliculata*, commonly known as apple snail or golden apple snail, is originating from South America but has become a globally distributed invasive species, listed among the top 100 worst alien invaders (Lowe  et al.  2000). This fresh water snail feeds on aquatic plants causing damages particularly in rice fields throughout Southeast Asia. Along with its role in agriculture *P. canaliculata* is known to be an intermediate host for the rat lungworm *Angiostrongylus cantonensis* (Lv  et al.  2009) and causes the potentially fatal disease of eosinophilic meningitis in humans (Pien  and  Pien  1999). It is also an important food source in some south Asian countries. Despite of this, the complete genome of this snail has not been sequenced, with only limited genetic studies conducted to date (Yang  et al.  2016; Liu  et al.  2018). A better understanding of the glycosylation mechanism of *Pomacea canaliculata* can provide insights into the snail-parasite interactions, which can not only aid in the development of effective control measures but also help to minimize its impact on crop production and human health.

*Crassostrea gigas*, also known as the Pacific oyster, is native to the Pacific coast of Asia. This bivalve mollusc has been introduced and cultivated in various parts of the world due to its high commercial value as a food source (Robinson  et al.  2005; Carrasco  and  Barón  2010; Meistertzheim  et al.  2013). These filter feeders extract suspended particles from the water column, providing not only nutrition but also promoting ecosystem health by improving water quality through the removal of excess nutrients. Pacific oysters have significant economic importance, being a highly prized seafood delicacy consumed in many countries. However, their extensive cultivation and aquaculture operations have environmental and ecological impacts (Herbert  et al.  2016). Genome sequencing data made *Crassostrea gigas* important also as a model organism (Zhang  et al.  2012). So far, not much is known about its glycosylation machinery.

Glycans play important roles in various physiological processes such as cell adhesion, communication, and recognition, as well as immune response. They are also important factors in the interaction between the organism and its environment, including interactions with pathogens and symbionts (Varki  2017; Cummings  2019). A better understanding of glycosylation of molluscs can have implications in various fields such as, nutrition and biomedicine. Different glycans structures may impact the nutritional quality of molluscs and the production of important bioactive compounds (Meng  et al.  2019; Prados-Castaño  et al.  2024). Last but not least, T-synthase can be used in medical industry for drug and vaccine development. T-synthases from different origins are already used for metabolic engineering of *Escherichia coli*, thus, enabling the bacteria to produce synthetic human milk oligosaccharides (Derya  et al.  2020; Hu  et al.  2022). Especially infant gut microbiota benefit from those glycans (Zhang  et al.  2022; Selvamani  et al.  2023). Further studies must be done to exactly evaluate the role of T-synthases in molluscs and their potential industrial applications.

After identifying and characterising the first T-synthase form mollusc origin (Zemkollari  et al.  2023), we present here two novel T-synthases, from the two different species *Pomacea canaliculata* and *Crassostrea gigas* including some molecular modelling aspects. Although nothing is known about the biological role of this enzyme in molluscs, identification of novel carbohydrate active enzymes and detailed knowledge of O-glycosylation processes in molluscs will help to gain a better understanding of their fascinating adaptation and survival abilities.

## Results

### Identification, cloning and expression of T-synthases from *Pomacea canaliculata* and *Crassostrea gigas*

**Fig. 1:**
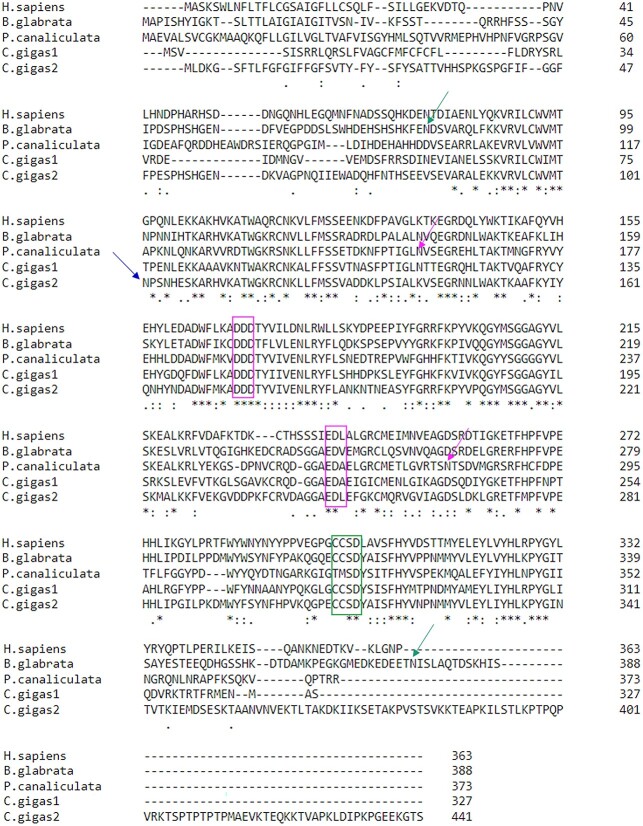
Sequence alignment of T-synthases from *H. sapiens* (NP_064541.1), *B. glabrata* (QXN57605.1), *P. Canaliculata* (WNA08525.1), *C. gigas1* (XP_011437048.2) and *C. gigas2* (WNA08526.1). Conserved domains are showed in magenta boxes (1st and 2nd box). The CCSD domain is showed in a green box (3rd box). Putative N-glycosylation sites are indicated by arrows (green, first and last, for *B. glabrata*, magenta, 2nd and 4th for *P. canaliculata* and blue, 3rd, for *C. gigas2*) (^*^ fully conserved residues, residues with strongly similar properties, residues with weakly similar properties, - indicates gap).

The sequences of both T-synthases were obtained by performing a blastP search using the *Biomphalaria glabrata* T-synthase sequence (QXN57605.1) as a template. In *Pomacea canaliculata*, we got a number of candidates. From the sequence analysis of the first 6 sequences (ranked lowest to highest E-value) we chose the sequence XP_025093750.1 because the first sequence was too long (XP_025094227.1) and the other 4 sequences had significant gaps. The resulting enzyme, Pc_T-synthase, showed sequence homology with previously characterized T-synthases from other species, with 49.61% amino acid similarity to *Homo sapiens*, 43.75% to *Drosophila melanogaster*, 42.32% to *Caenorhabditis elegans*, and 43.63% to *Biomphalaria glabrata* (Table 1). The identified coding sequence is 1,122 bps long. For *Crassoststrea gigas*, we followed the same logic. The first sequence was chosen based on E-value, percentage of similarity and sequence analysis compared with the Pc_T-synthase and the second one with the Bg_T-synthase. The identified sequences are: a 984 bps long sequence (Cg1_T-synthase, XP_011437048.2), and a 1,266 bps long sequence (Cg2_T-synthase, XP_034309164.1). The first sequence (Cg1_T-synthase) showed amino acid sequence similarities with previously described T-synthases, with 52.53% similarity to *Homo sapiens*, 51.54% to *Drosophila melanogaster*, 50.00% to *Caenorhabditis elegans*, and 50.74% to *Biomphalaria glabrata*. The second sequence (Cg2_T-synthase) showed slightly more similarity to previously described T-synthases, with 54.45% amino acid similarity to *Homo sapiens*, 54.09% to *Drosophila melanogaster*, 49.58% to *Caenorhabditis elegans*, and 66.56% to *Biomphalaria glabrata* (Table 1). The selected genes encoded T-synthases that were 373 amino acids long (Pc_T-synthase), 327 amino acids long (Cg1_T-synthase), and 421 amino acids long (Cg2_T-synthase) (Fig. 1). We successfully expressed the coding sequences in Sf9 cells. We found no difference in yields nor in activity rates when the HisTag was N-terminally or C-terminally fused to the enzyme, so we chose to work with the C-terminal HisTag constructs. The cloned sequences were submitted to NCBI GenBank with the accession numbers OQ974926.1 (*Pomacea canaliculata*, new protein accession number for Pc_T-synthase WNA08525.1) and OQ974927.1 (*Crassostrea gigas*, new protein accession number for Cg2_T-synthse WNA08526.1).

**Table 1:** Sequence homology comparison of the two novel mollusc T-synthases with previously identified ones from other species.

	Pc_T-synthase	Cg1_T-synthase	Cg2_T-synthase
*B. glabrata*	43.36%	50.74%	66.56%
*H. sapiens*	49.61%	52.53%	54.45%
*D. melanogaster*	43.75%	51.54%	54.09%
*C. elegans*	42.32%	50.00%	49.58%

### Core 1 β-1,3-galactosyltransferase activity

The activity of the Sf9 cell lysate expressing T-synthase and a control Sf9 cell lysate was assessed, revealing that the Pc_T-synthase exhibited core 1 β-1,3-galactosyltransferase activity towards the pNP-α-GalNAc substrate (Fig. S1). Among the *Crassostrea gigas* β-1,3-galactosyltransferases, only the Cg2_T-synthase displayed activity. Although the Cg1_T-synthase contained conserved motifs and exhibited sequence similarities with previously described T-synthases, it did not transfer galactose to the pNP-α-GalNAc substrate. Notably, Cg2_T-synthase demonstrated higher transfer rates than the Pc_T-synthase (at least 20% more) despite having lower expression levels (Fig. S2).

### Sequence analysis

The β-1,3-galactosyltransferases responsible for core 1 synthesis are Golgi type II transmembrane proteins: Pc_T-synthase: amino acids 17–39 comprise the transmembrane helix; Cg1_T-synthase: amino acids 12–31 comprise the transmembrane helix; Cg2_T-synthase: amino acids 7–29 comprise the transmembrane helix; predicted by TMHMM Server v. 2.0. Pc_T-synthase and Cg1_T-synthase each have two putative N-glycosylation sites, while Cg2_T-synthase has one. Orbitrap MS analysis of the peptides confirmed that these sites were occupied by typical insect cell N-glycan structures (mostly H3N2F and H3N3F) (Fig. S3). The protein sequences feature conserved glycosyltransferase motifs, including DDD and EDV (or EDA/EDL), which are involved in divalent cation and UDP-sugar binding (Malissard  et al.  2002). The CCSD motif is believed to be a hallmark feature of all T-synthases and related proteins, and it is conserved in all vertebrate and invertebrate T-synthases known until now (Fig. S4). In Pc_T-synthase, this motif is modified to TMSD. All known invertebrate T-synthases have seven cysteine residues in their catalytic domain, but none in their transmembrane domain (except the T-synthase form *Drosophila melanogaster*). From the mollusc T-synthases only the one from *Biomphalaria glabrata* seems to obey this unwritten rule. Pc_T-synthase has three cysteines in the catalytic domain and one in the N-terminal (cytosolic) domain. Cg1_T-synthase has seven cysteine residues in the catalytic domain and three in the transmembrane domain, while Cg2_T-synthase has only six conserved cysteine residues in the catalytic domain (Fig. 1).

### Effect of mutations on Pc_T-synthase activity

To investigate the reason for the lower activity rates of Pc_T-synthase compared to Cg2_T-synthase, we examined the sequence and hypothesized that the modified CCSD motif may be responsible. As CCSD is considered a hallmark for all T-synthases, we believed it could be critical for enzyme activity. To test this, we introduced two amino acid mutations, T320C and M321C, to restore the conserved motif and expressed the resulting mutant, mPc_T-synthase, in Sf9 insect cells. However, contrary to our expectations, the introduced mutations not only failed to improve enzyme activity, but actually decreased it (Fig. S5). This suggests that the CCSD motif is a structural motif and has no implications in the activity of the enzyme. It is also possible that the artificial addition of these two cysteines might have further consequences that abolish the activity of the enzyme.

### Specificity determination of the T-synthase

To determine the activity of Pc_T-synthase and Cg2_T-synthase, Sf9 cell lysates expressing these enzymes were compared to a control lysate using HPLC. Only the lysates expressing T-synthase displayed high levels of activity, indicating that the β-1,3-galactosyltransferase activity was generated solely by the expressed enzymes from *Pomacea canaliculata* and *Crassostrea gigas* and not by a host cell enzyme. By comparing the chromatograms of the activity reactions with those of the standard pNP-α-GalNAc and standard pNP-α-GalNAc-β-1,3-Gal, the product of the activity reaction was identified as pNP-α-GalNAc-β-1,3-Gal. This is consistent with the product of *Biomphalaria glabrata* T-synthase (Zemkollari  et al.  2023).

**Fig. 2:**
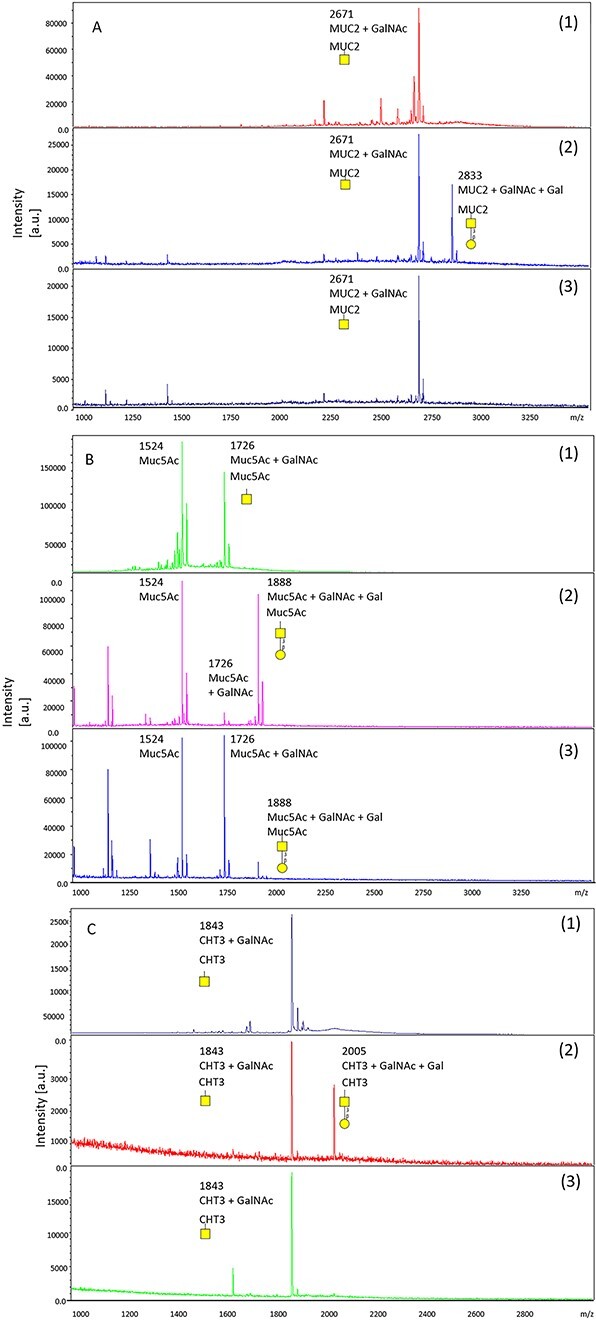
MALDI-TOF analysis of the transfer of Gal to (a) α-GalNAc-Muc2, (b) α-GalNAc-Muc5Ac, and (c) α-GalNAc-CHT3. (1) α-GalNAc-peptide, (2) α-GalNAc-peptide incubated with Cg2_T-synthase and (3) α-GalNAc-peptide incubated with Pc_T-synthase.

To explore the substrate specificity of the expressed enzymes, pNP-labelled monosaccharides such as pNP-α-Gal, pNP-α-Glc, pNP-β-GalNAc, pNP-β-Gal, pNP-β-Glc, and pNP-β-GlcNAc were tested, but none of them was a suitable substrate for the mollusc T-synthases. It was determined that the only functional substrate was the monosaccharide GalNAc in the α-position, although an α-GalNAc structural element alone was not sufficient. Peptides such as α-GalNAc-Muc2, α-GalNAc-Muc5Ac, and α-GalNAc-CHT3, which had been previously monoglycosylated by UDP-N-acetyl-α-D-galactosamine: polypeptide-N-acetylgalactosaminyl-transferase from *Biomphalaria glabrata* were tested. Pc_T-synthase did not galactosylate α-GalNAc-Muc2 or α-GalNAc-CHT3, but appeared to be suitable for galactosylating α-GalNAc-Muc5Ac. Cg2_T-synthase was able to galactosylate all three of them: α-GalNAc-Muc2, α-GalNAc-Muc5Ac, and α-GalNAc-CHT3 (Fig. 2) (Table 2).

**Table 2:** Comparison of the three mollusc T-synthase.

		Bg_T-synthase	Pc_T-synthase	Cg2_T-synthase
Optimal pH		6.0	6.5	7.0
Salt buffer		MES	MES	MES
Cation		✔	✔	✔
Best		Mn^2+^	Mn^2+^	Mn^2+^
Opt. Incubation Temp.		37 °C	20 °C	20 °C
α-GalNAc -peptides	CHT3	✔ **✕** **✕**	**✕** **✕** ✔	✔ ✔ ✔
	Muc2			
	Muc5Ac			
N-glycosylation sites		2	2	1
Cys Residues	Cytosolic	0 0 7	1 0 3	0 0 6
	TM			
	Catalytic			

### Biochemical properties of the enzymes

**Fig. 3:**
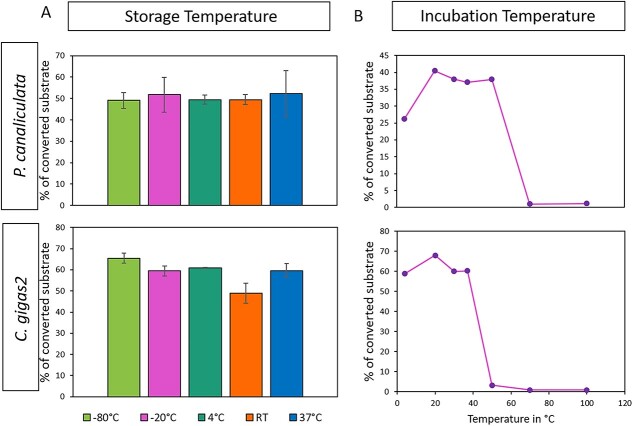
Effects of (a), storage temperature and (b), incubation temperature on the activity of Pc_T-synthase (first row) and Cg2_T-synthase (second row).

**Fig. 4:**
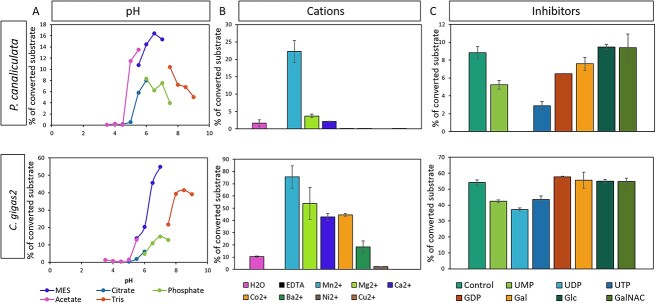
Effect of (a), pH, (b), cations and (c), inhibitors on the activity of Pc_T-synthase (first row) and Cg2_T-synthase (second row).

The T-synthases’ activity was found to be relatively stable upon storage at temperatures ranging from −80 °C to 37 °C for 24 h (Fig. 3a). The best temperature for incubation was 20 °C for both of them (Fig. 3b). However, the addition of 5%–15% methanol decreased the activity of Cg2_T-synthase by half, while the addition of 5%–15% acetonitrile progressively reduced the activity. On the other hand, the addition of up to 15% glycerol did not affect the activity of either enzyme. For Pc_T-synthase, the presence of 5%–15% methanol, acetonitrile, and glycerol significantly decreased its activity (Fig. S6). The pH optimum for Pc_T-synthase was found to be 6.5 when tested with MES buffer, while Cg2_T-synthase showed maximum transfer rates with MES buffer at pH 7.0 (Fig. 4a). Phosphate buffer is not a good choice for either enzyme. It may have an inhibitory effect by blocking the UDP-binding site of the enzyme (Krajewska  and  Zaborska  1999). The enzymatic reaction of both T-synthases was found to be dependent on divalent cations, as observed in the standard activity assay without any cations and in the presence of 20 mM EDTA, Mn^2+^, Mg^2+^, Ca^2+^, Co^2+^, Cu^2+^, Ni^2+^, or Ba^2+^. The presence of EDTA abolished the transfer activity of both enzymes, while the presence of Mn2+ resulted in the highest transfer rates. For Pc_T-synthase, the preferred order of cations was found to be Mn^2+^ > Mg^2+^ > Ca^2+^, while for Cg2_T-synthase, the order was Mn^2+^ > Mg^2+^ > Co^2+^ > Ca^2+^ (Fig. 4b). The addition of 5 nmols of UMP, UDP, UTP, GDP, and galactose was found to inhibit the activity of Pc_T-synthase. The addition of UMP and UTP decreased the activity by 30% and 75%, respectively, while UDP completely inhibited the activity. Addition of GDP and galactose reduced the activity by 25% and 13%, respectively. However, glucose and N-acetylgalactosamine had no effect on the activity. For Cg2_T-synthase, the addition of 5 nmols of UMP, UDP, or UTP slightly decreased the activity, with reductions of 23%, 31%, and 16%, respectively. The addition of GDP, galactose, glucose, and N-acetylgalactosamine had no effect on the enzyme’s activity (Fig. 4c). All biochemical properties of the mollusc T-synthases are summarised in Table 2.

### Predicted 3D structure of the T-synthases

**Fig. 5:**
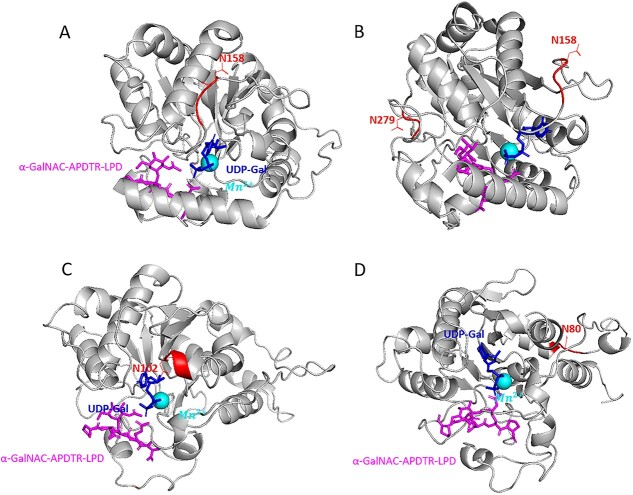
Overall structure of the modelled mollusc enzymes in complex with UDP-Gal (sticks in dark blue, in the middle of each image), acceptor glycopeptide (sticks in magenta, located in the lower part of the figure) and manganese ion (light blue ball) highlighting the putative N-glycosylation sites in red with the Asn position. (a) Pc_T-synthase front view, (b) Pc_T-synthase side view, (c) Cg2_T-synthase front view and (d) Bg_T-synthase front view.

**Fig. 6:**
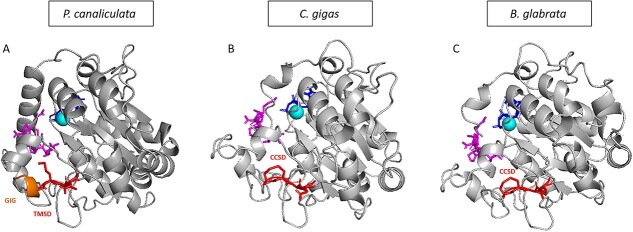
Overall structure of the modelled mollusc enzymes in complex with UDP-Gal (sticks in dark blue close to the manganese), glycopeptide (sticks in magenta at the left side) and manganese ion (light blue ball) highlighting the TMSD (in red) and GIG (in orange) motifs for (a) Pc_T-synthase and the CCSD (in red in the lower part of the figures) motif for (b) Cg2_T-synthase and (c) Bg_T-synthase.

To predict the 3D structure, we used Alphafold2 (Fig. S7). Alphafold2 is a very reliable tool for protein structure prediction. The alignment of the Alphafold2 prediction of the *Drosophila melanogaster* T_synthase structure with the X-ray crystallography model (7Q4I) has an atom-positional root-mean-square deviation (RMSD) of 1.390. For Pc_T-synthase, the residues 1–44 (Alphafold2 predicts an α-helix) and residues 45–62 (Alphafold2 predicts a linker) are not shown due to the very low per-residue confidence score (pLDDT). The same goes for residues 359–371 at the other end of the protein. For the residues 63–81 Alphafold2 predicts an α-helix and the residues 82–101 a linker that do not have a very high pLDDT value compared to the rest of the structure but still higher than the parts which are not shown. For Cg2_T-synthase and Bg_T-synthase we followed the same rule so only residues 51–340 and 50–337 are shown on the 3D model respectively. The additional α-helix (63–81) is specific to *Pomacea canaliculata* (Fig. S8). Alphafold2 does not predict an α-helix in this position for any of the other T-synthases (vertebrate and invertebrate). It is positioned right at the place where the substrate is located. After modelling of the substrate and cofactor, based on the *D. melanogaster* T-synthase crystal structure, it appears that an aspartate (D75) in this α-helix is pointing directly to the manganese ion (Fig. S9a). There are also three other aspartates and three glutamates in this α-helix, resulting in a highly negative charge of the enzyme. Consequently, the interaction with the manganese ion becomes very strong. This conformation might result in an “arm” formed by the supposed helix very close to the entrance of the catalytic site, which may hinder the substrate to bind easily. In the model, the position of the α-helix interferes with the putative position of the substrate peptide, suggesting that conformational changes need to take place to accommodate it, and offering an explanation to the reduced activity of the enzyme. In contrast, Cg2_T-synthase and Bg_T-synthase have a histidine pointing at the manganese ion and have predicted structures that are intrinsically more flexible at the binding site of the substrate peptide (Fig. S9b and c). The putative N-glycosylation sites are shown in Fig. 5. For Pc_T-synthase, the first N-glycosylation site (N158) is located on top of the catalytic site whereas the second N-glycosylation site (N279) is at the back of the enzyme with the asparagine facing outwards (Fig. 5a and b). The only putative N-glycosylation site of Cg2_T-synthase (N102) is also close to the catalytic site and the asparagine is predicted to be facing towards it (Fig. 5c). Being this close to the active site, the glycans might be involved in the activity by facilitating the interaction with the acceptor and donor substrates. For Bg_T-synthase the first N-glycosylation (N80) site is located at the back of the catalytic site with the asparagine facing completely outwards. While the second N-glycosylation site is in the region 338–388 that is not shown due to the low pLDDT value (Fig. 5d). The cysteines in the CCSD domain form disulphide bridges in Cg2_T-synthase and Bg_T-synthase causing the formation of a loop (Fig. 6b and c). To compensate the lack of cysteines, the Pc_T-synthase has a GIG motif in front of the TMSD motif. Glycine is the most flexible amino acid so the GIG motif enables the formation of the loop despite the lack of cysteines (Fig. 6a). These observations lead to the suggestion that the CCSD motif might be more a structural motif than a functional one.

## Discussion

Here we present the identification and characterisation of two T-synthases from mollusc origin. These enzymes play a crucial role in understanding the process of O-glycosylation in the phylum Mollusca. The T-synthase transfers a galactose from UDP-galactose to an α-bound GalNAc in the polypeptide chain, resulting in the formation of the core 1 O-glycan (T-antigen). While mammalian enzymes require the molecular chaperone Cosmc for proper folding and activity, invertebrate enzymes appear to fold correctly without the need for such a chaperone, as no Cosmc homolog has been found in their genomes or is necessary for folding or enzymatic activity. This has been shown for *Caenorhabditis elegans* (Ju  et al.  2006) and *Bombyx mori* (Morio  et al.  2023). In our studies on mollusc T-synthases (from *Biomphalaria glabrata*, *Pomacea canaliculata* and *Crassostrea gigas* (Zemkollari  et al.  2023 and this study) no homolog of Cosmc was detectable or necessary either. Previous reports have shown that the stem region can act like a chaperone during the folding of human and bovine β-1,4-galactosyltransferase (Boeggeman  et al.  2003). It is possible that invertebrate T-synthases maybe rely on the stem region for folding in the absence of a specific molecular chaperone. Of course, the currently available data (one insect, one worm, three molluscs) is still very limited for drawing conclusions about invertebrates in general.

The novel mollusc enzymes were expressed using an insect cell-Baculovirus expression system, known for its simplicity and speed. The similar glycosylation patterns between insects and molluscs establish this system as the optimal choice for mollusc enzyme expression. Furthermore, in our previous study we showed that expression of *Biomphalaria glabrata*_T-synthase in yeast (*Komagatella phaffi*) and in *Escherichia coli* resulted in inactive enzymes (Zemkollari  et al.  2023).

The putative N-glycosylation sites of the recombinant mollusc T-synthases expressed in Sf9-cells were occupied by typical insect cell N-glycans. This leads to the suggestion that these enzymes may also be N-glycosylated in their native environment and that the selection of an expression system which is able to create similar glycan structures is of high importance.

Cysteine residues in the catalytic domain of T-synthases are conserved across different evolutionary groups. Vertebrate enzymes have six conserved cysteine residues, while invertebrate enzymes have a seventh cysteine residue in the catalytic domain. However, *Drosophila melanogaster’s* T-synthase deviates from this pattern, having one cysteine residue in the transmembrane domain. Among the mollusc T-synthases, only the T-synthase of *Biomphalaria glabrata* adheres to this pattern, with seven cysteine residues in the catalytic domain. In contrast, Pc_T-synthase has only three cysteine residues in the catalytic domain and one in the N-terminal (cytosolic) domain, and Cg2_T-synthase has only six conserved cysteine residues in the catalytic domain. Interestingly, Cg1_T-synthase, which lacks core 1 β-1,3-galactosyltransferase activity, has three cysteine residues in the transmembrane domain and seven in the catalytic domain. The rest of the structure shows conserved motifs and high sequence similarity compared to the other three working mollusc T-synthases, especially with Pc_T- synthase (56.59%) (Fig. 1). So, this difference in cysteine residues might be the reason for the inactivity of this construct.

Two of the cysteines missing in Pc_T-synthase belong to the CCSD motif. This motif is conserved throughout all vertebrate and invertebrate enzymes known until now. We attempted to improve the activity of Pc_T-synthase by mutating the TMSD sequence to CCSD, assuming it would enhance the enzyme’s activity. Surprisingly, not only did this mutation fail to improve the activity, but it actually decreased it. This indicates that the CCSD motif is not essential for the activity of the T-synthase or that the artificial introduction of the two cysteines have implications in the structure of the enzyme. The necessities in the conformation also seem to be fulfilled in Pc_T-synthase by the presence of an additional GIG motif. Besides the missing CCSD motif, another remarkable feature of the Pc_T-synthase is the fact that AlphaFold2 predicts an additional helix, which has not yet been detected in this form in any other of the known T-synthases.

In addition to sharing structural resemblances with previously described T-synthases the mollusc T-synthases also share biochemical similarities with them. They have a maximum activity at pH 6.5 (Pc_T-synthase) and pH 7.0 (Cg2_T-synthase). They both require divalent cations for activity as addition and EDTA completely abolishes their activity (Table 2). The activity of Pc_T-synthase was inhibited by the addition of 5 nmols of UMP, UDP, UTP, GDP, and galactose. Especially, the addition of UMP decreased the activity by 30%, UTP by 75%, and UDP completely inhibited the activity. The addition of GDP and galactose reduced the activity by 25% and 13%, respectively. However, the presence of glucose or N-acetylgalactosamine had no impact on the enzyme’s activity. For Cg2_T-synthase, the addition of 5 nmols of UMP, UDP, or UTP resulted in a slight decrease in activity, with reductions of 23%, 31%, and 16%, respectively. While the addition of GDP, galactose, glucose, and N-acetylgalactosamine did not affect the enzyme’s activity. Biochemical properties of previously described T-synthases are unknown so comparing them with our mollusc T-synthases is not possible. Nevertheless, in the activity assays described, the human, rat, *Drosophila melanogaster* and *Bombyx mori* T-synthases were active using MES buffer pH 6.8 and *Caenorhabditis elegans* T-synthase MES buffer pH 6.5, while all of them required 20 mM MnCl_2_ (Ju  et al.  2002a; Ju  et al.  2002b; Ju  et al.  2006;Yoshida  et al.  2008; Morio  et al.  2023).

None of pNP- α-Gal, pNP- α-Glc, pNP-β-GalNAc, pNP-β-Gal, pNP- β-Glc, and pNP- β-GlcNAc proved to be suitable substrates for the mollusc T-synthases. It was determined that the only functional substrate was GalNAc in the α-position, although the presence of an α-GalNAc structural element alone was not sufficient for activity. Even different GalNAc-peptides were not suitable for each of the enzymes. Pc_T-synthase was unable to galactosylate α-GalNAc-Muc2 or α-GalNAc-CHT3, but galactosylated α-GalNAc-Muc5Ac. Cg2_T-synthase demonstrated the ability to galactosylate all three substrates, namely α-GalNAc-Muc2, α-GalNAc-Muc5Ac, and α-GalNAc-CHT3 (Fig. 2). This difference in α-GalNAc-peptide specificity might be correlated with the position of the GalNAc. Neighbouring amino acids might influence the recognition of α-bound GalNAc and so modulate the activity of the enzymes (Perrine  et al.  2009).

T-synthases are important modifiers of O-glycan structures. They can be used for the biosynthesis of glycan structures which have positive effects on our microbiome by preventing the binding of pathogens (Derya  et al.  2020; Zhang  et al.  2022; Selvamani  et al.  2023). Due to the fact, that invertebrate enzymes do not need the help of the molecular chaperone Cosmc for proper folding, they may be in the future an interesting alternative for the metabolic engineering of expression systems established for the production of medically relevant oligosaccharides.

In this study and recently (Zemkollari  et al.  2023) we have identified and characterised in total three T-synthases from mollusc origin (two freshwater snails, one marine oyster). Some information is available from other invertebrates such as worms (*Caenorhabditis elegans*) (Ju  et al.  2006) or insects (*Drosophila melanogaster* and *Bombyx mori*) (Yoshida  et al.  2008; Morio  et al.  2023). However, even this small sample of examples shows clearly, that although when there are some similarities with vertebrates, the glycosylation machinery of invertebrates is much more complex and diverse. We are far away from understanding the whole glycosylation process in invertebrates in all its aspects. In particular investigation on the large phylum of molluscs will for sure uncover some biological surprises in the future. Unexpected solution strategies may contribute to the fact that this phylum is so incredibly successful in colonising all habitats on the planet.

## Materials and methods

### Materials

Restriction enzymes, T4 ligase, Q5 DNA polymerase and OneTaq DNA Polymerase were all purchased from New England Biolabs (Frankfurt, Germany). Plasmid purification and Gel clean-up kits were purchased from Macherey-Nagel (Düren, Germany). The g-Block gene fragment was synthetized by Integrated DNA Technologies (Leuven, Belgium). All primers were commercially synthetized by Integrated DNA Technologies or Sigma Aldrich (Vienna, Austria).

Chemically competent *Escherichia coli* (High Efficiency) cells (New England Biolabs, Frankfurt, Germany), DH10 Multibac YFP *E. coli* cells (Geneva Biotech, Geneva Switzerland) and *Spodoptera frugiperda* cells (Sf9, ATCC CRL-1711, Manassas Virginia) were cultivated in IPL41 medium (HyClone Cytiva—Vienna, Austria) were used.

### Identification of the gene sequence

The protein sequence of the T-synthase from *Pomacea canaliculata* and *Crassostrea gigas* were obtained by performing a blastP search, using the sequence of the *Biomphalaria glabrata* T-synthase sequence (MW720711). The sequences to be cloned were chosen based on similarities with other already known invertebrate T-synthases. They were codon optimized for insect cells and synthetized by Integrated DNA Technologies (Leuven, Belgium).

### Cloning and expression of the synthetic sequences

The optimized gene sequences were cloned into the pACEBac1 vector. Three distinct constructs for each gene, were created: C-terminus His Tag construct, N-Terminus His Tag construct and a soluble construct (without the transmembrane domain) with C-terminal 6xHisTag. A Bac-To-Bac Baculovirus expression system was used to express the enzymes in Sf9 insect cells as described at (Zemkollari  et al.  2023). To introduce the point mutations in the Pc_T-synthase the following primers were used: 5′ CCT TTA CGA GCA CCG TTG GTG TCG and 5′ AATTGGA TGC TGT AGCGATTACAGTATAACC.

### Activity assay of the recombinant T-synthase

The activity of the recombinant T-synthase was measured in a total volume of 20 μL reaction mixture containing: 50 mM MES pH 6.5, 20 mM MnCl_2_, 200 μM UDP-Gal (Sigma-Aldrich, Vienna, Austria), 1 mM pNP-α-GalNAc, 2 mM ATP and 5 μL (roughly estimated to contain 0.5 μg/μL (Pc_T-synthase) and 0.01 μg/μL (Cg2_T-synthase) enzyme) enzyme at 37 °C for 2 h. 3×108 transfected Sf9 cells were used for protein extraction in 4 mL of 400 mM MES buffer. The cells were lysed using Ultraturrax and the clear supernatant was used for activity tests. The reaction was terminated by cooking the sample at 100 °C for 5 min and analysed by HPLC on a reversed phase C18 column 4 × 250 mm, 5 μm (Thermo Scientific, Vienna, Austria) in 0.1 M ammonium acetate pH 6.0 (solvent A) applying a linear gradient with solvent B (acetonitrile in water 50:50) from 5 to 50% in 30 min at a flow rate of 1 mL/min with a detection at 280 nm.

### Biochemical parameters

For the analysis of the biochemical parameters the standard assay conditions were modified as follows. For the determination of cation requirements, the assay was carried out without any cations or in presence of 10 mM EDTA, Mn^2+^, Mg^2+^, Ca^2+^, Ba^2+^, Co^2+^, Cu^2+^ and Ni^2+^. To determine the optimum Mn^2+^, the concentration of MnCl_2_ was varied from 0–30 mM. The determination of enzyme stability and pH optimum was done according to (Zemkollari  et al.  2023). Substrate specificity was tested with the artificial substrates pNP- α-Gal, pNP- α-Glc, pNP-β-GalNAc, pNP-β-Gal, pNP- β-Glc and pNP-β-GlcNAc, benzyl-α-GalNAc and the α-GalNAc containing peptides PTTTPITTTTTVTP**T**(GalNAc)PTPTGTQTK (α-GalNAc-Muc2), GTTP**S**(GalNAc)PVPTTSTTSAP (α-GalNAc-Muc5Ac) and APPAHPGP**T**(GalNAc)PGYRPAPG (α-GalNAc-CHT3) under standard assay conditions (the amino acid where the GalNAc is attached is shown in bold). The peptides were commercially synthesised, monoglycosylated by UDP-N-acetyl-α-D-galactosamine: polypeptide-N-acetylgalactosaminyl-transferase from *Biomphalaria glabrata* and purified on HPLC according to (Taus  et al.  2013). Inhibition experiments were carried out by adding 5 nmols of UMP, UDP, UTP, GDP, Gal, GalNAc or Glc to the standard incubation assay. All quantitative values were calculated from the area of HPLC chromatograms and each assay was carried out at least in duplicate with appropriate controls.

### (Glyco)peptide analysis

The sample was digested in-gel. The proteins were S-alkylated with iodoacetamide and digested with Trypsin (Promega). The digested samples were loaded on a nanoEase C18 column (nanoEase M/Z HSS T3 Column, 100 Å, 1.8 μm, 300 μm X 150 mm, Waters) using 0.1% v/v formic acid as the aqueous solvent. A gradient from 1% B (B: 80% v/v acetonitrile, 0.1% v/v formic acid) to 40% B in 50 min was applied, followed by a 10 min gradient from 40% B to 95% B that facilitates elution of large peptides, at a flow rate of 6 μL/min. Detection was performed with an Orbitap MS (Exploris 480, Thermo) equipped with the standard H-ESI source in positive ion, DDA mode (= switching to MSMS mode for eluting peaks). MS-scans were recorded (range: 350–1,200 Da) and the 20 highest peaks were selected for fragmentation. Instrument calibration was performed using Pierce FlexMix Calibration Solution (Thermo Scientific).

The possible glycopeptides were identified as sets of peaks consisting of the peptide moiety and the attached N-glycan varying in the number of HexNAc units (N), hexose (H), pentose (X), sialic acid (S) and deoxyhexose (F) residues. The theoretical masses of these glycopeptides were determined with a spread sheet using the monoisotopic masses for amino acids and monosaccharides. Manual glycopeptide searches were made using FreeStyle 1.8 (Thermo). For the quantification of the different glycoforms the peak intensities of the deconvoluted spectra were compared. The analysis files analysed using PEAKS, which is suitable for performing MS/MS ion searches.

### Matrix assisted laser desorption ionisation—Time of flight (MALDI-TOF)

Mass Spectrometry MALDI-TOF MS analysis was carried out on an Autoflex Speed MALDI-TOF (Bruker Daltonics, Germany) equipped with a 1,000 Hz Smartbeam.II laser in positive mode using α-cyano-4-hyroxycinnamic acid as matrix (1% w/v in 65% v/v acetonitrile solution). For crystallization 1 μL of an 1:40 dilution of the samples was spotted on the plate, air dried, covered by 1 μL of matrix solution and again air dried. Spectra were processed with the manufacturer’s software (Bruker Flexanalysis 3.3.80).

### Molecular modelling

A structural model for Pc_T-synthase, Cg2_T-synthase and Bg_T-synthase was made using ColabFold v1.5.2-patch: AlphaFold2 using MMseqs2 (Mirdita  et al.  2022). While the quality of the model seems quite good, we emphasize that these models remain predictions, that will need to be validated experimentally. PyMOL.Ink was used to visualise the pdb file from ColabFold. *Drosophila melanogaster’s* T-synthase crystal structure (7Q4I) was used as a template to determine the positions of the substrates and the cofactor. The coordinates of UDP-Gal, Mn^2+^ and α-GalNAc-APDTRP were taken also from 7Q4I crystal structure after aligning the catalytic domain of the modelled enzymes.

## Supplementary Material

Supplementary_material_20240209_Glycobiology_final_cwae013

## Data Availability

The sequences identified in this study are available on NCBI.
