# Supplementary material for: Molecular cloning, characterisation and molecular modelling of two novel T-synthases from mollusc origin
Source: Glycobiology. 2024 Feb 17;34(4):cwae013. doi: 10.1093/glycob/cwae013 (PMC11005171; doi:10.1093/glycob/cwae013)
Supplement: Supplementary_material_20240209_Glycobiology_final_cwae013 [file supplementary_material_20240209_glycobiology_final_cwae013.pdf]

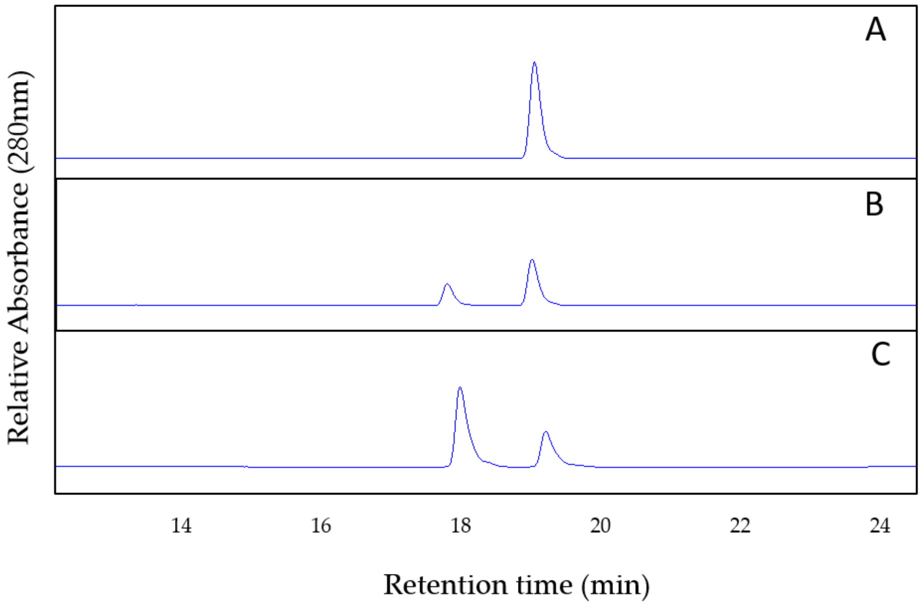

Figure S1. HPLC analysis of the transfer of Gal to pNP-α-GalNAc. (A) pNP-α-GalNAc incubated with cell lysate expressing an inactive GalT (negative control), (B) pNP-α-GalNAc incubated with Pc_T-synthase, (C) pNP-α-GalNAc incubated with Cg2_T-synthase.

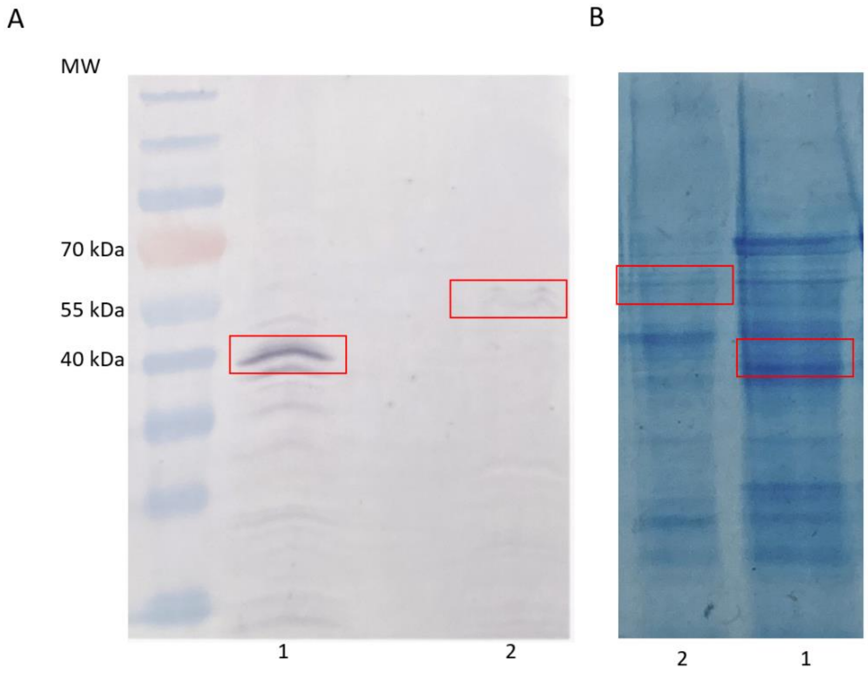

Figure S2. (A) Western blot analysis cell lysates expressing the recombinant T-synthases from (1) *P. canaliculata* (43kDa) and (2) *C. gigas* (50 kDa) and (B) Coomassie staining of purified (HisTag antibody linked with protein A/G agarose beads) (1) *C. gigas* (50 kDa) and (2) *P. canaliculata* (43kDa). The read squares indicate the bands for the enzymes.

Glycan composition	Monoisotopic mass	%
H3N2F	2442,105	40,15
H3N3F	2645,181	26,04
H3N2	1959,796	13,78
H5N2	2283,901	5,49
H3N3	2162,875	2,93
H7N2	2608,007	1,08
H6N2	2315,913	1,05
H3N4F	2512,014	0,92
H4N2	2121,85	0,50
H2N2	1797,743	0,48

H-Hexose, N-N-acetylhexosamine, F-Fucose

Figure S3. MS/MS analysis of glycosylation sites of Pc_T-synthase and Cg2_T-synthase. Top 10 most abundant N-glycans found.

```
H.sapiens          IGKETFHPFVPEHHLIKGYLPRTFWYWNYNYYPPVEGPGCCSDLAVSFHYVDSTTMYELE    320
R.norvegicus       TGKETFHPFVPEHHLIKGYLPKTFWYWNYNYYPPVEGPGCCSDIAVSFHYVDSTTMYELE    328
C.elegans          DGHHRFMPFVPEHHLSPGHVDPKFWFWQYTYYPMDQGPTCCSDYAVSFHYVNPNLMYVLE    340
D.melanogaster     NGRGRFFPFVPEHHLIPSHTDKKFWYWQYIFYKTDEGLDCCSDNAISFHYVSPNQMYVLD    336
B.mori             MRRGRFFPFVPQDHLFPN-KDKGFWYWSYIYYPSDEGLDCCSDHAVTFHYVNPQQMYVFD    313
B.glabrata         LGRERFHPFVPEHHLIPDILPPDMWYWSYNFYPAKQGQECCSDYAISFHYVPPNMMYVLE    327
C.gigas2           LGRETFMPFVPEHHLIPGILPKDMWYFSYNFHPVKQGPECCSDYAISFHYVNPNMMYVLE    329
P.canaliculata     MGRSRFHCFDPETFLFGGYP---DWYYQYDTNGARKGIGTMSDYSITFHYVSPEKMQALE    340
                     :  *  * *: .*  .      *::.*      :*    ** :::****    *  ::
```

Figure S4. Alignment of all known T-synthases showing the conserved CCDS domain in black box. (* fully conserved residues, : residues with strongly similar properties, . residues with weakly similar properties, - indicates gap). (*H. sapiens* NP_064541.1, *R. norvegicus* NP_001385639.1, *C. elegans* NP_499293.2, *D. melanogaster* NP_609258.1, *B. glabrata* QXN57605.1, *B. mori* XP_021202995.1, *P. canaliculata* WNA08525.1 and *C. gigas* WNA08526.1).

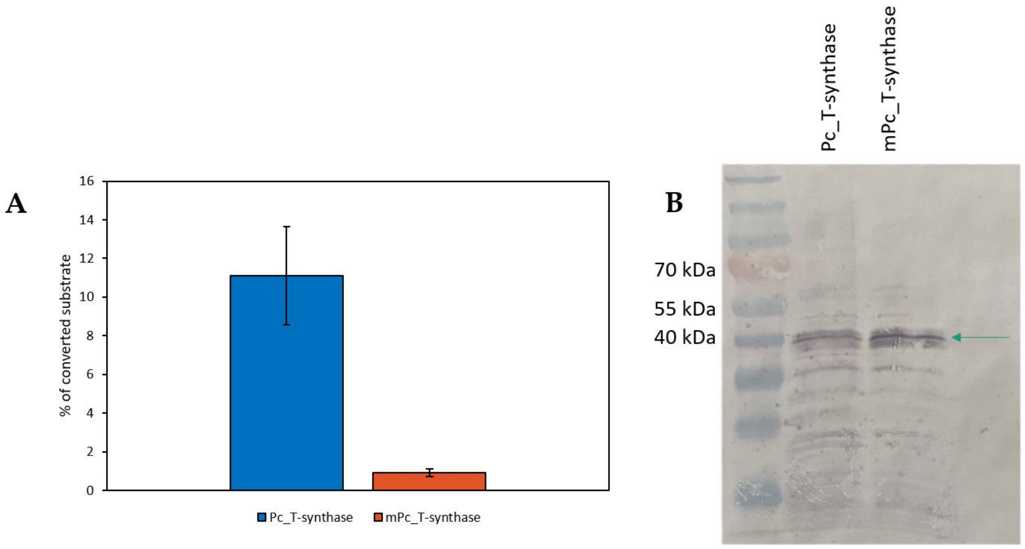

Figure S5. Activity levels of (A), Pc_T-synthase and mPc_T-synthase and (B), Western blot analysis cell lysates expressing Pc_T-synthase and mPC_T-synthase. The green arrow shows the bands for the enzymes.

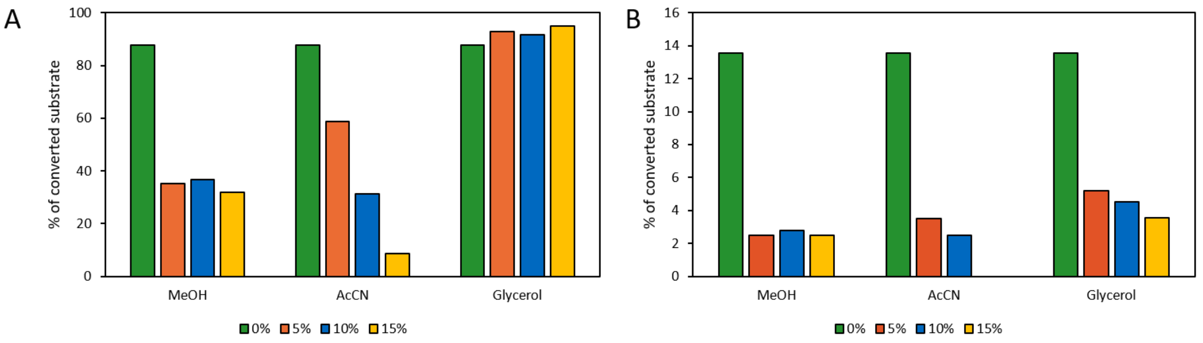

Figure S6. Effects of methanol, acetonitrile and glycerol on the activity of (A) Cg2_T-synthase and (B) Pc_T-synthase.

```
H.sapiens       QPNV----LHNDPHARHSD--DNG-------QNHLEG-QMNFNADSSQHKDENTDIAENL    83
R.norvegicus    QPNM----LHNDPHARHSD--DSG-------HNHLKG-QMDFNADSSQHKDENTDVAENL    91
C.elegans       LRAN-RGDPQVDEHDHAHGNDPHGDEEVDDHHANFAPVQ-FHSNNSSHSHDGESLIADEV    100
D.melanogaster  MPY--------DGHRHGDVNDAHH------SHDMMELTGPEQDVGTHEHVHENSTIAERL    96
B.mori          WPA--------YRESLRDL----D------RHPIVNIVD--HGSDEPAHKDEDRSIADEL    74
B.glabrata      RHFSSSGYIPDSPHSHGENDFVEG------P----DDSLSWHDEHSHSHKFENDSVARQL    87
C.gigas2        PGFIFGGFFPESPHSHGENDKVAG------P----NQIIEWADQHFNTHSEEVSEVARAL    89
P.canaliculata  PNFV--GR---PSGVIGDEAFQRDDHEAWDRSIERQGPGIMLDIHDEHAHHDDVSEARRL    105
                                                          .             *  :
```

Figure S7. Alignment of all known invertebrate T-synthases (*C. elegans* NP_499293.2, *D. melanogaster* NP_609258.1, *B. glabrata* QXN57605.1, *B. mori* XP_021202995.1, *P. canaliculata* WNA08525.1 and *C. gigas* WNA08526.1) showing the sequence that corresponds to Pc_T-synthase's α-helix (residues 63-81) in black box in comparison with the vertebrate enzymes from human (NP_064541.1) and rat (NP_001385639.1) (* fully conserved residues, : residues with strongly similar properties, . residues with weakly similar properties, - indicates gap).

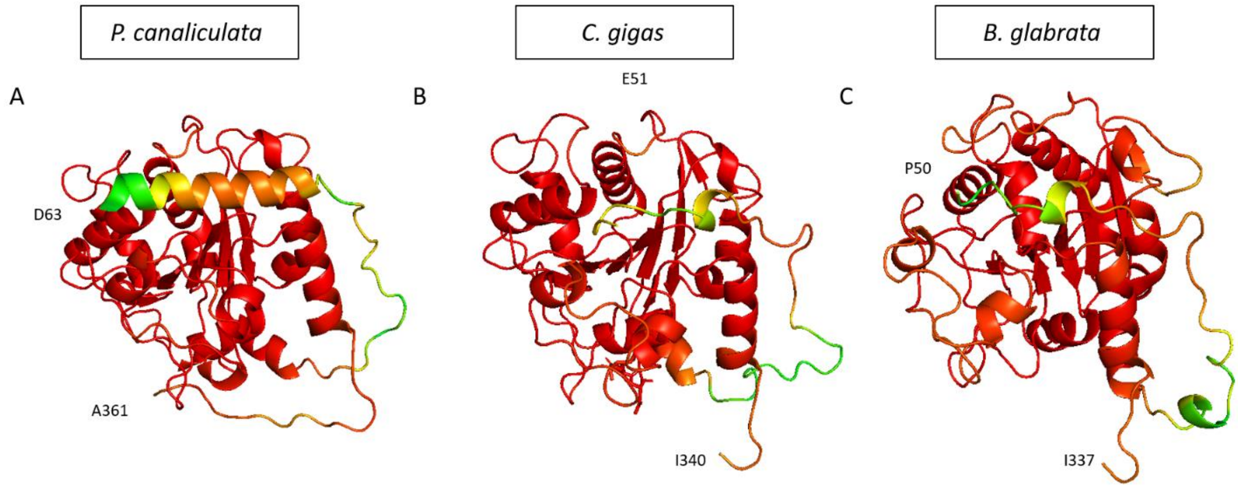

Figure S8. Overall structure of the modelled mollusc enzymes (A), Pc_T-synthase, (B), Cg2_T-synthase and (C), Bg_T-synthase.

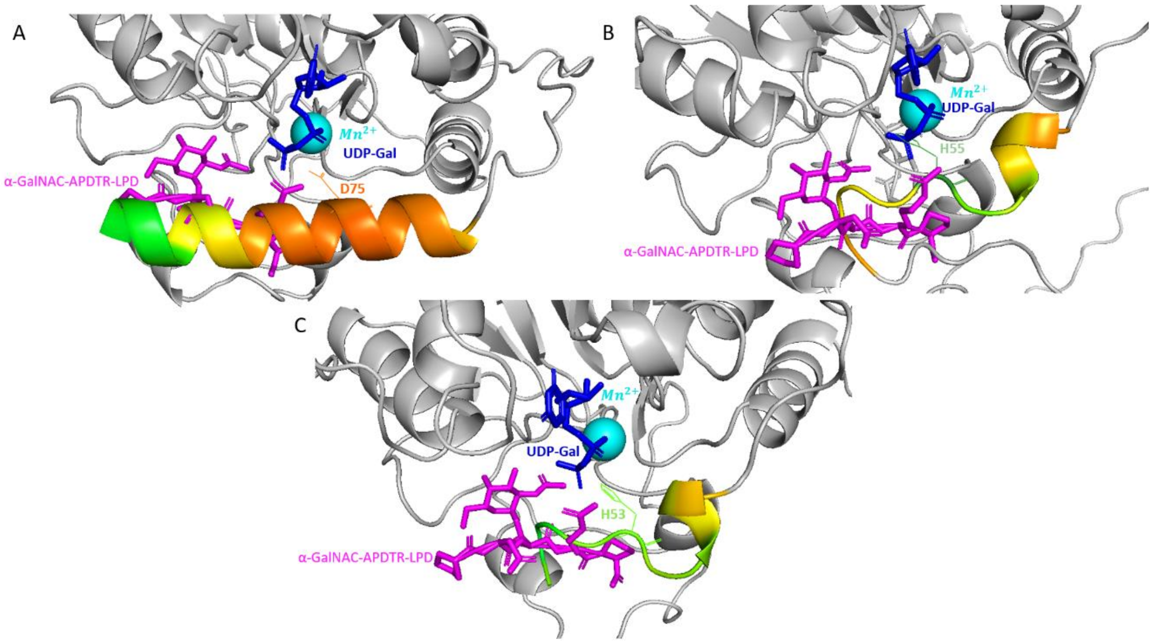

Figure S9. Enzymes in complex with UDP-Gal (sticks in dark blue), glycopeptide (sticks in magenta) and manganese ion (light blue). (A) Pc_T-synthase, (B) Cg2_T-synthase and (C) Bg_T-synthase, focused on the steam region (residues 63-81, Pc_T-synthase).
